# Supplementary figures and images for: Microbial Metabolic Redundancy Is a Key Mechanism in a Sulfur-Rich Glacial Ecosystem
Source: mSystems. 2020 Aug 4;5(4):e00504-20. doi: 10.1128/mSystems.00504-20 (PMC7406229; doi:10.1128/mSystems.00504-20)

**Supplementary Material – Figure S1. BFP16 Spring location.**

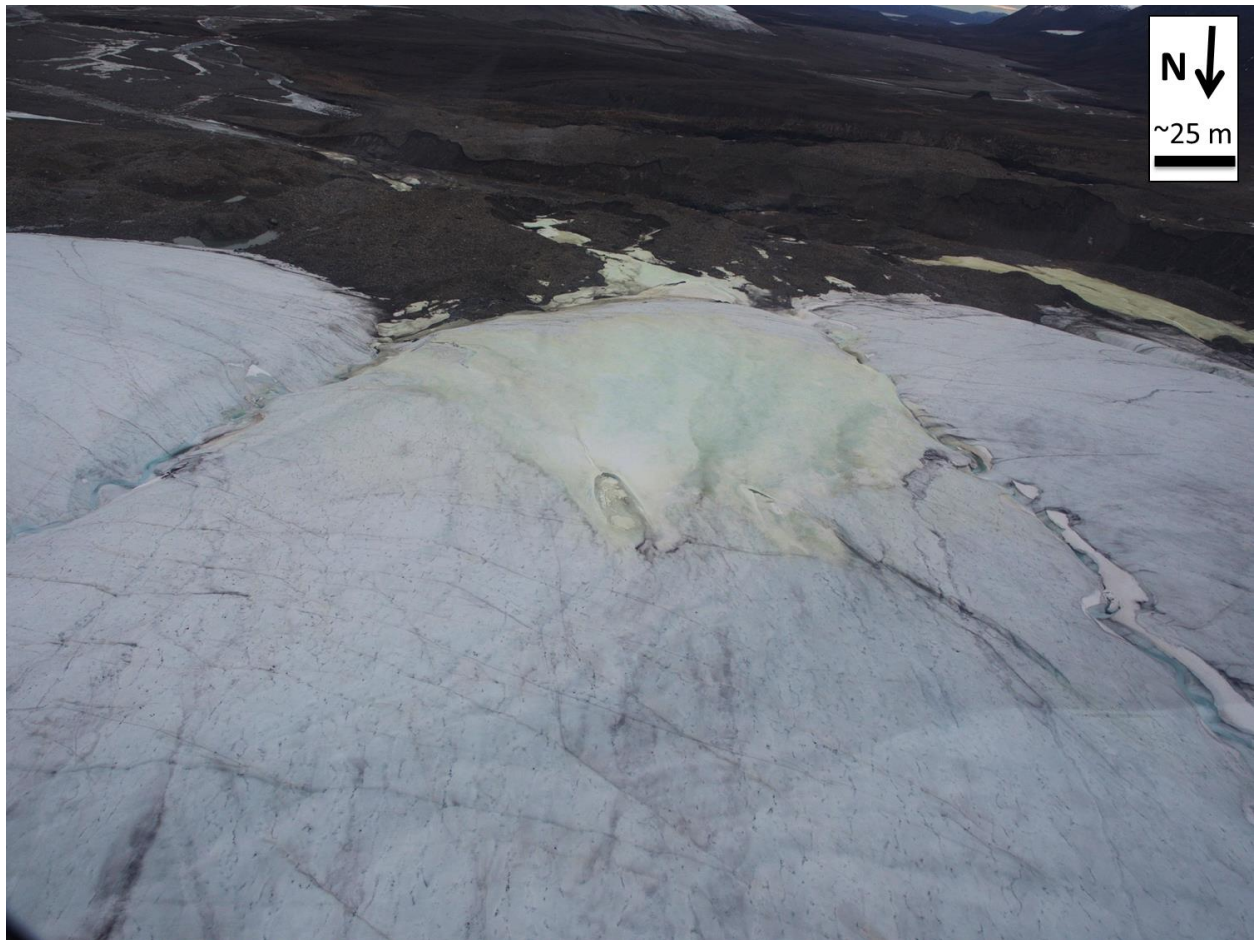

Supplement: FIG S1 [file mSystems.00504-20-sf001.pdf]

Supplementary Material - Figure S2. Sample type examples

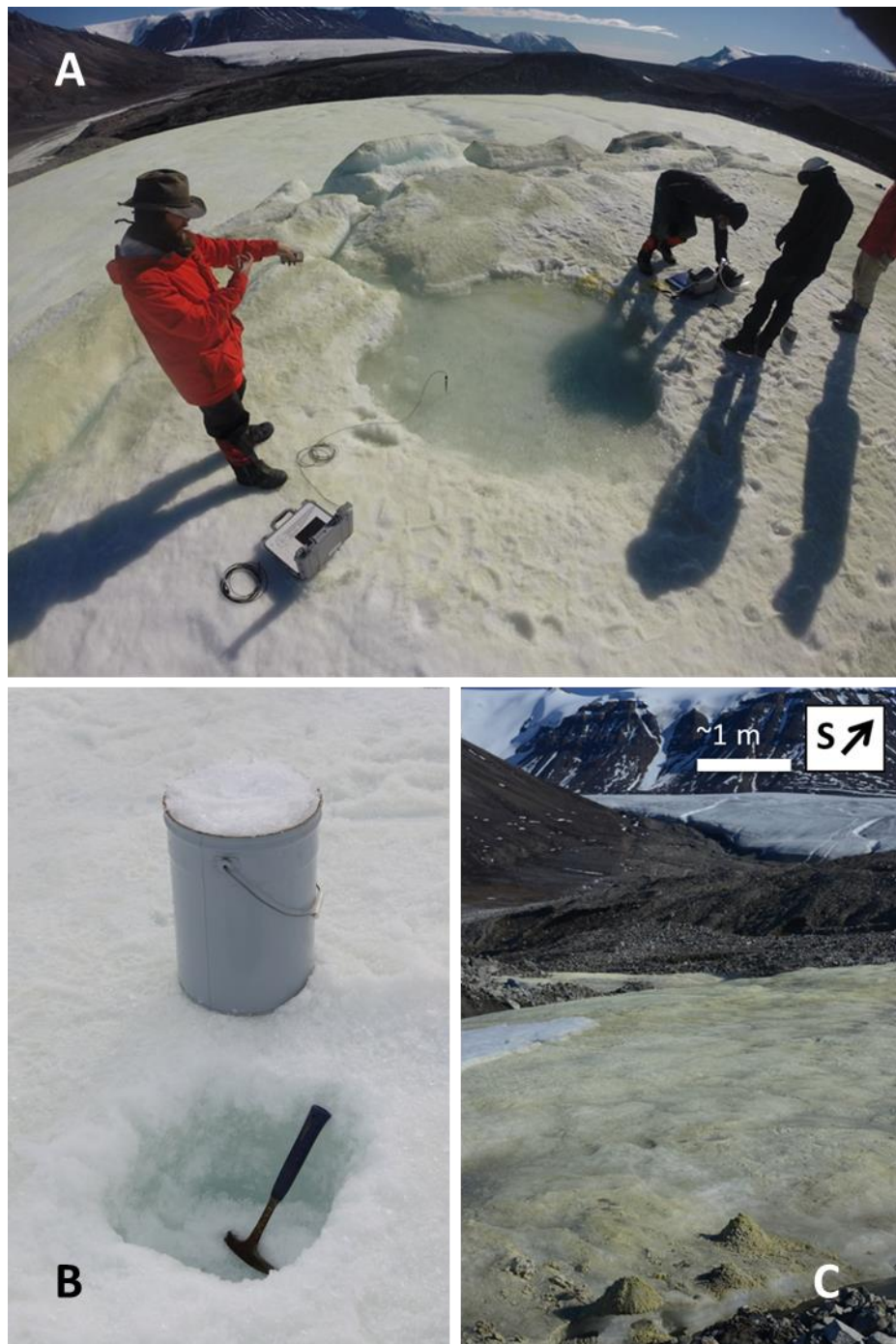

Supplement: FIG S2 [file mSystems.00504-20-sf002.pdf]

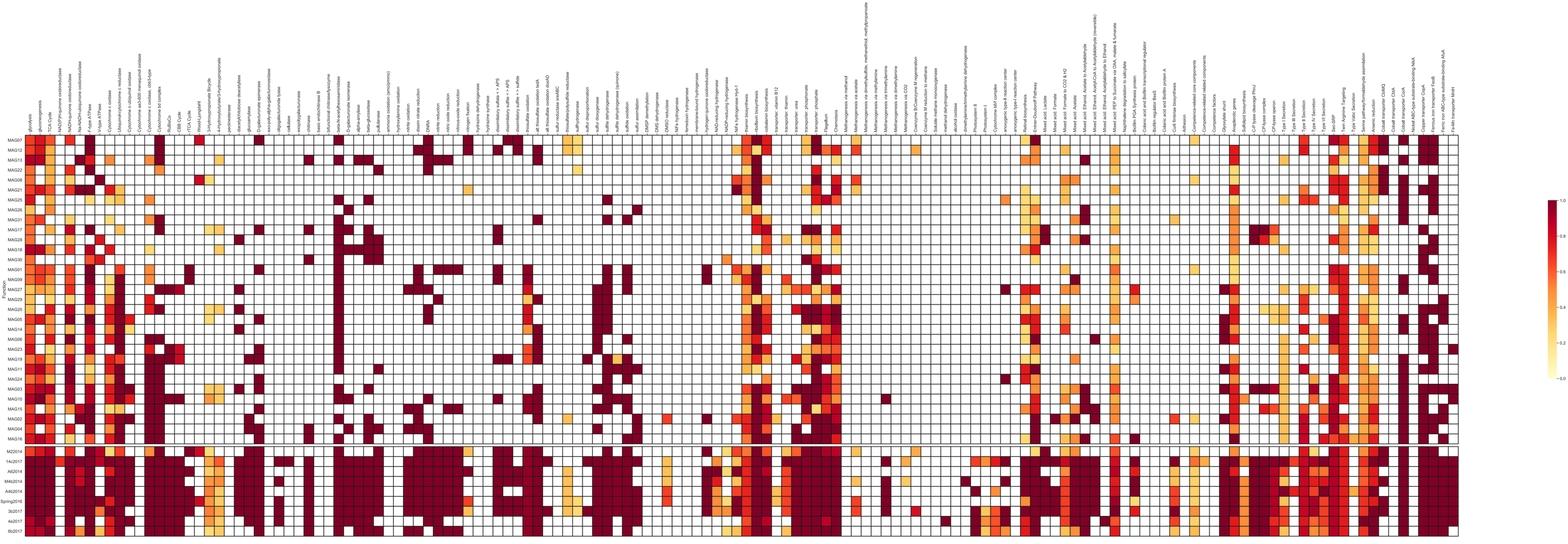

Supplement: FIG S3 [file mSystems.00504-20-sf003.pdf]
